# Novel plasma biomarkers improve discrimination of metabolic health independent of weight

**DOI:** 10.1038/s41598-020-78478-w

**Published:** 2020-12-07

**Authors:** Stephen Ellison, Jawan W. Abdulrahim, Lydia Coulter Kwee, Nathan A. Bihlmeyer, Neha Pagidipati, Robert McGarrah, James R. Bain, William E. Kraus, Svati H. Shah

**Affiliations:** 1 Department of Anesthesiology, Duke University Medical Center, Durham, NC USA; 2 Duke Molecular Physiology Institute, Duke University School of Medicine, 300 North Duke St, Durham, NC 27701 USA; 3 Division of Cardiology, Department of Medicine, Duke University School of Medicine, Durham, NC USA

**Keywords:** Biomarkers, Prognostic markers, Cardiology

## Abstract

We sought to determine if novel plasma biomarkers improve traditionally defined metabolic health (MH) in predicting risk of cardiovascular disease (CVD) events irrespective of weight. Poor MH was defined in CATHGEN biorepository participants (n > 9300), a follow-up cohort (> 5600 days) comprising participants undergoing evaluation for possible ischemic heart disease. Lipoprotein subparticles, lipoprotein-insulin resistance (LP-IR), and GlycA were measured using NMR spectroscopy (n = 8385), while acylcarnitines and amino acids were measured using flow-injection, tandem mass spectrometry (n = 3592). Multivariable Cox proportional hazards models determined association of poor MH and plasma biomarkers with time-to-all-cause mortality or incident myocardial infarction. Low-density lipoprotein particle size and high-density lipoprotein, small and medium particle size (HMSP), GlycA, LP-IR, short-chain dicarboxylacylcarnitines (SCDA), and branched-chain amino acid plasma biomarkers were independently associated with CVD events after adjustment for traditionally defined MH in the overall cohort (*p* = 3.3 × 10^−4^–3.6 × 10^−123^), as well as within most of the individual BMI categories (*p* = 8.1 × 10^−3^–1.4 × 10^−49^). LP-IR, GlycA, HMSP, and SCDA improved metrics of model fit analyses beyond that of traditionally defined MH. We found that LP-IR, GlycA, HMSP, and SCDA improve traditionally defined MH models in prediction of adverse CVD events irrespective of BMI.

## Introduction

Claiming more lives in 2015 than the combined mortality of the second and third leading causes, cancer and chronic lower respiratory disease, respectively, cardiovascular disease (CVD) remains the leading cause of morbidity and mortality within the United States^1^. Obesity, a known risk factor for a variety of negative health effects including CVD and adverse CVD-related events^2^, remains a major public health challenge; in fact, the age-adjusted percentage of adults greater than 20 years old with obesity in the US increased from 22.9% in 1988–1994 to 37.8% in 2013–2014^3^. The relationship between obesity and CVD has been partially attributed to the increased prevalence of intermediate risk factors including insulin resistance, type 2 diabetes mellitus (DM), dyslipidemia, and hypertension. However, the underpinnings remain incompletely elucidated and the heterogeneity of prevalence of these risk factors in obesity demonstrates the complexity of the relationship^4^.

Metabolic health (MH) has traditionally been defined by the presence or absence of a cluster of intermediate CVD risk factors known as metabolic syndrome^5^, while medical literature has also classically defined being overweight and having poor metabolic health as synonymous^6^. Metabolic syndrome is a known contributor to the epidemic of CVD^7^, however, a growing body of evidence suggests that weight is a poor metric of metabolic health^4,8^ even though obesity continues to be reported as a “major determinant of metabolic syndrome^9^”.

While intermediate CVD risk factors seen in metabolic syndrome are important, perhaps the most important metric for defining metabolic health should be its ability to associate with hard cardiovascular endpoints. Poor metabolic health as defined by metabolic syndrome is associated with increased risk of major adverse cardiovascular events (MACE) in the overall population^7^, but evidence suggests the risk of MACE is not well correlated with BMI category^10^, often as a result of underestimating the risk of those patients who have components of metabolic syndrome but do not meet full criteria^11,12^.

In order to more effectively target interventions to improve metabolic health and prevent adverse CVD events, there is a need for better markers of metabolic health beyond traditionally defined MH that are predictive regardless of BMI. Plasma biomarkers reflect dysregulated systemic and tissue-specific metabolism, serving as downstream read-outs of genetic, transcriptomic and proteomic variation, and thus may serve as these better markers. In fact, recent studies in our lab using metabolomic profiling have identified a number of biomarkers that are associated with obesity, insulin resistance, DM, CVD, and response to interventions, including low-density lipoprotein (LDL-P)^13–15^, lipoprotein-insulin resistance (LP-IR)^16^, GlycA^17^, sum of medium plus small high-density lipoprotein-particle subclass (HMSP)^18^, short-chain dicarboxylacylcarnitines (SCDA)^19,20^, and branched-chain amino acids (BCAA)^20–22^. Thus, we hypothesized that these novel plasma biomarkers can improve predictive capabilities for incident adverse CVD events across BMI categories and are better markers of metabolic health than traditional measures which often rely heavily on BMI.

## Methods

CATHGEN biorepository participants (n > 9300), a follow-up cohort (> 5600 days) comprising participants undergoing evaluation for possible ischemic heart disease.

### Study population

Individuals undergoing cardiac catheterization between 2001 and 2010 for the indication of possible ischemic heart disease at Duke University Medical Center provided written consent and enrolled in the CATHGEN (CATHeterization GENetics) biorepository (n > 9300) as previously described^23^. For this follow-up cohort, longitudinal annual follow-up for recurrent events, hospitalizations, and vital status has been performed and confirmed through the National Death Index and the Social Security Death Index for > 5600 days from time of CATHGEN enrollment. CATHGEN is approved by the Duke University School of Medicine Institutional Review Board in accordance with the Declaration of Helsinki (Durham, NC, USA). Prior to administration of supplemental heparin, 50 mL of blood was collected at the time of arterial puncture from fasting, consented participants. Samples were immediately cooled to 4 °C, processed to separate plasma, and then frozen to − 80 °C. Clinical data including demographics, medical comorbidities, angiographic data, quantitation of left ventricular ejection fraction (LVEF), and laboratory data were collected. For this study, participants were excluded if they were cardiac transplant recipients (n = 201), had undergone cardiac catheterization for the indication of pulmonary hypertension (n = 94), had a history of congenital heart disease (n = 27), had missing BMI (n = 1), had no longitudinal follow-up data (n = 8), had missing biomarker data (n = 115), or had incomplete data necessary for calculation of traditionally defined MH (n = 226).

Race was classified as white or non-white. LVEF was obtained by ventriculogram at the time of cardiac catheterization or, if ventriculography was not performed, by an echocardiogram or cardiac magnetic resonance image (MRI). Coronary artery disease was determined by the physician performing the cardiac catheterization, and defined as present if a stenosis ≥ 50% was present in any major epicardial coronary vessel. For this study, the primary endpoint was defined as time-to-event for all-cause mortality or MI from CATHGEN enrollment.

### Definition of BMI categories and traditionally defined poor MH

Individuals were classified into three categories based on BMI at time of enrollment: lean (BMI < 25 kg/m^2^), overweight (BMI 25–30 kg/m^2^), or obese (BMI ≥ 30 kg/m^2^). Individuals were categorized as having traditionally defined poor MH if they had at least two out of four criteria for poor MH at baseline enrollment, and considered to have traditionally defined good MH if they met 0–1 criteria (modified slightly from the Adult Treatment Panel III (ATPIII) guidelines^24^ given lack of waist circumference data in CATHGEN): (1) fasting triglycerides (TG) ≥ 150 mg/dL, (2) HDL < 40 mg/dL in men or < 50 mg/dL in women, (3) history of diabetes, insulin use, fasting glucose ≥ 100 mg/dL, or HOMA-IR > 5.13, (4) blood pressure medication use or history of patient reported hypertension. Homeostatic model assessment of insulin resistance was calculated as follows: HOMA-IR = (fasting insulin in (µUI/mL) * glucose (mM)/22.5^21,25^. A HOMA-IR threshold of > 5.13 (i.e., the 90th percentile) was selected and consistent with prior work^21^.

### Metabolomic profiling

Lipoprotein particle concentrations, sizes, and subclasses as well as GlycA, LP-IR, and creatinine were measured as previously described^17,20,26^. For this study, we used a composite sum of medium plus small HDL-particle subclass (diameters ≤ 9.4 nm, termed HMSP) because we have previously reported HMSP as more strongly associated with incident CVD events than individual HDL components^17^. GlycA is an inflammatory biomarker that consists of the NMR signal from the *N*-acetyl methyl groups of the *N*-acetylglucosamine residues on enzymatically glycosylated acute phase proteins (primarily α1-antichymyotrypsin, α1-acid glycoprotein, haptoglobin, α1-antitrypsin, and transferrin)^17^. The LP-IR score is a measure of insulin resistance derived using the weighted sum of six NMR derived lipoprotein parameters^16^; the multiplex validated score ranges from 0 to 100 (where 100 is the most insulin resistant).

For measurement of acylcarnitines and amino acids, targeted metabolic profiling was performed using flow-injection, tandem mass spectrometry (MS/MS) as previously reported^27^. Values below the lower limits of quantitation (LLOQ) were reported and analyzed as “0,” while plasma biomarkers with > 25% of values below the LLOQ were not analyzed.

Principal components analysis (PCA) with varimax rotation was used for reduction of metabolic data into identifiable factors, with those factors having an eigenvalue of ≥ 1.0 being retained, and plasma biomarkers with a factor load of ≥ 0.4 being reported as comprising a factor as previously described^20,27,28^. In our prior work, a PCA-derived factor composed of SCDA (glutaryl carnitine (C5-DC), 3-hydroxy-cis-5-octenoyl carnitine or hexenedioyl (C6:1-DC/C8:1-OH), octenedioyl carnitine (C8:1-DC), adipoyl carnitine (C6-DC), methylmalonyl carnitine or succinyl carnitine (Ci4-DC/C4-DC), 3-hydroxy-decanoyl carnitine or suberoyl carnitine (C10-OH/C8-DC), 3-hydroxy-dodecanoyl carnitine or sebacoyl carnitine (C12-OH/C10-DC), and citrulline) is predictive of incident CVD events. Thus, that PCA factor was used in this study. Prior work has also shown a PCA-derived factor of BCAA and two aromatic amino acids is associated with CAD and adverse CVD events; thus, that PCA factor was also used in this study (composed of leucine/isoleucine, valine, phenylalanine, tyrosine, and methionine)^27^. These PCA-factors for SCDA and BCAA are used as measures for the composite of SCDA and BCAA in this study.

### Statistical methods

Baseline clinical and demographic characteristics were compared between individuals with and without traditionally defined poor MH using *t*-tests and chi-squared tests for continuous and categorical variables, respectively. Incident events were defined as all-cause mortality or MI (adjudicated by a central process within the Duke Databank for Cardiovascular Disease). Cox proportional hazards models were used to examine the independent and additive effects of plasma biomarkers on time-to-event, accounting for traditionally defined poor MH. Specifically, we tested three models for time-to-event, each adjusted for BMI category (lean, overweight, obese) in the full sample: (1) traditionally defined poor MH; (2) each individual plasma biomarker alone (LDL-P, LP-IR, GlycA, HMSP, BCAA, and SCDA), adjusted for traditionally defined poor MH; and (3) a full model inclusive of traditionally defined poor MH and all plasma biomarkers jointly. We also stratified the data by BMI category, and tested the same models to understand the potential heterogeneity of effects of the relationship of traditionally defined poor MH and novel plasma biomarkers across BMI categories. To evaluate medications as a confounder, we performed sensitivity analyses on a subset of individuals with available medication data, where time-to-event models were adjusted for statin use. Statin use was determined using EHR admission medications, i.e. an individual was considered to be on a statin if they were on a statin at admission either 365 days prior to or 10 days after the index cardiac catheterization. Finally, while our primary goal was to identify better markers of metabolic health compared to traditionally defined MH parameters (using association with incident events as the metric to determine “better”), in sensitivity analyses, we also created models that included traditional CVD risk factors (age, sex, race, smoking, ejection fraction, LDL-C, creatinine, family history of CAD, and presence/absence of CAD). The assumption of proportional hazards was tested for each model; when violated, we included a term in the model with a time-varying coefficient and compared the resulting inference to inference based on the original model. We used the Akaike Information Criterion (AIC) and area under the receiver operating characteristic curve (AUC) to compare model fit between different models and determine the incremental discriminative capability of novel plasma markers. AIC analyses were restricted to individuals who had data needed for all models of traditionally defined MH and for all biomarkers (n = 3222).

## Results

Table 1 shows baseline characteristics of the overall study population (n = 8671 individuals) stratified by both metabolic health and BMI categories; 1921 (22.2%) individuals were classified as lean, 3068 individuals (35.4%) were overweight, and 3682 individuals (42.5%) were obese. Obese individuals tended to be younger than overweight or lean individuals (59.2 ± 11.2 years vs. 62.8 ± 11.7 years vs. 64.1 ± 13.1 years, respectively) and had greater prevalence of races other than white. Approximately 45% of obese and lean individuals were female compared with only 30% of overweight individuals.

**Table 1:** Baseline characteristics of the CATHGEN study population stratified by traditionally defined MH and BMI category.

	Stratified by BMI category							
	Overall		Lean (BMI < 25)		Overweight (BMI 25–30)		Obese (BMI ≥ 30)	
	Good MH	Poor MH	Good MH	Poor MH	Good MH	Poor MH	Good MH	Poor MH
n (%)	2486 (28.7)	6185 (71.3)	858 (44.7)	1063 (55.3)	947 (30.9)	2121 (69.1)	681 (18.5)	3001 (81.5)
Age (years)	61.4 (12.5)	61.6 (11.7)	62.5 (13.5)	65.4 (12.7)**	62.0 (11.8)	63.1 (11.6)*	59.1 (12.0)	59.3 (11.0)
Female (%)	40.7	37.3*	47.3	43.1**	30.8	29.7	46.3*	40.8*
BMI (kg/m^2^)	27.7 (6.2)	31.1 (7.4)**	22.2 (2.1)	22.6 (2.0)**	27.3 (1.4)	27.6 (1.4)**	35.2 (6.1)	36.7 (6.6)**
White (%)	78.8	73.2**	80.3	78.1	81.2	77.8*	73.6	68.2*
Hypertension (%)	42.4	90.3**	39.0	90.2**	43.7	88.3**	44.6	91.8**
Diabetes (%)	8.6	67.3**	8.0	57.9**	9.2	62.8**	8.5	73.7**
Glucose (mg/dL)	97.4 (21.8)	126.6 (53.7)**	95.7 (23.3)	117.6 (49.2)**	98.0 (20.2)	122.7 (50.0)**	98.7 (21.7)	132.5 (57.0)**
HOMA-IR	1.22 (1.30)	2.42 (2.65)**	0.84 (0.99)	1.50 (2.00)**	1.23 (1.34)	2.05 (2.25)**	1.70 (1.45)	3.03 (2.97)*
Smoking (%)	1112 (44.7)	2970 (48.0)*	412 (48.0)	530 (49.9)	422 (44.6)	1059 (49.9)*	278 (40.8)	1381 (46.0)*
LVEF%	57.0 (12.5)	54.4 (14.3)**	56.3 (13.0)	52.7 (15.9)**	57.4 (12.3)	54.2 (14.2)**	57.4 (12.3)	55.2 (13.8)**
CAD (%)	1229 (52.8)	4188 (70.2)**	384 (48.9)	740 (73.3)**	529 (58.6)	1541 (74.8)**	316 (49.5)	1907 (65.9)**
Family history of CAD (%)	744 (29.9)	2128 (34.4)**	247 (28.8)	343 (32.3)	284 (30.0)	730 (34.4)*	213 (31.3)	1055 (35.2)
HDL (mg/dL)	54.4 (18.0)	41.6 (13.0)**	59.5 (21.4)	44.3 (15.0)**	52.3 (15.7)	41.5 (12.5)**	50.9 (14.4)	40.8 (12.4)**
LDL (mg/dL)	106.7 (36.5)	100.4 (40.9)**	102.9 (34.9)	96.0 (39.8)*	106.7 (37.1)	101.3 (40.6)**	111.9 (37.2)	101.4 (41.3)**
Creatinine (mg/dL)	1.08 (1.03)	1.27 (1.34)**	1.11 (1.38)	1.36 (1.66)**	1.08 (0.80)	1.30 (1.37)**	1.06 (0.77)	1.22 (1.18)**
TG (mg/dL)	127.1 (92.5)	189.2 (193.8)**	106.9 (68.5)	151.1 (110.0)**	133.9 (92.9)	176.4 (141.8)**	144.1 (113.0)	211.0 (239.5)**
**Follow-up**								
MI (%)	110 (4.4)	453 (7.3)**	36 (4.2)	59 (5.6)	46 (4.9)	177 (8.3)**	28 (4.1)	217 (7.2)*
Death (%)	883 (35.5)	2710 (43.8)**	398 (46.4)	594 (55.9)**	300 (31.7)	935 (44.1)**	185 (27.2)	1181 (39.4)**
Time to death/MI (days)	2526 (1278)	2450 (1307)**	2305 (1287)	2231(1341)**	2539 (1228)	2438 (1268)**	2630 (1185)	2458 (1239)**

*p < 0.05, **p < 0.001 for comparison to good MH.

All continuous variables reported as mean (SD).

*MH* Traditionally defined metabolic health, *BMI* body mass index, *HOMA-IR* homeostatic model assessment-insulin resistance, *LVEF* left ventricular ejection fraction, *CAD* coronary artery disease, *HDL* high-density lipoprotein, *LDL* low-density lipoprotein, *TG* triglycerides, *MI* myocardial infarction.

Overall, there was a high prevalence of traditionally defined poor MH (71.3%). As expected, obese and overweight individuals had a greater prevalence of poor MH compared with lean individuals (81.5% vs. 69.1% vs. 55.3%), although the prevalence of poor MH was high even in the lean group. Concurrently, 18.5% of obese individuals were defined as having good MH by traditional parameters, further confirming the known disconnect between metabolic health and weight. The increasing prevalence of poor MH across increasing BMI categories was driven by all components of the metabolic health measure including higher prevalence of hypertension and diabetes, higher TG, and lower HDL. Interestingly, LDL was lower in poor MH individuals as compared with good MH individuals across each of the three BMI categories (96.0 vs. 102.9 mg/dL lean, 101.3 vs. 106.7 mg/dL overweight, 101.4 vs. 111.9 mg/dL obese, poor vs good MH individuals respectively).

In the overall cohort, individuals with poor metabolic health had a higher prevalence of a positive family history of CAD as compared with individuals with good metabolic health (34.4% vs. 29.9%, *p* < 0.001), which was also true in the overweight category (35.2% vs. 31.3%, *p* < 0.05). However, there were no significant differences in the lean and obese group.

### Association of BMI and traditionally defined poor MH with incident CVD events

**Figure 1:**
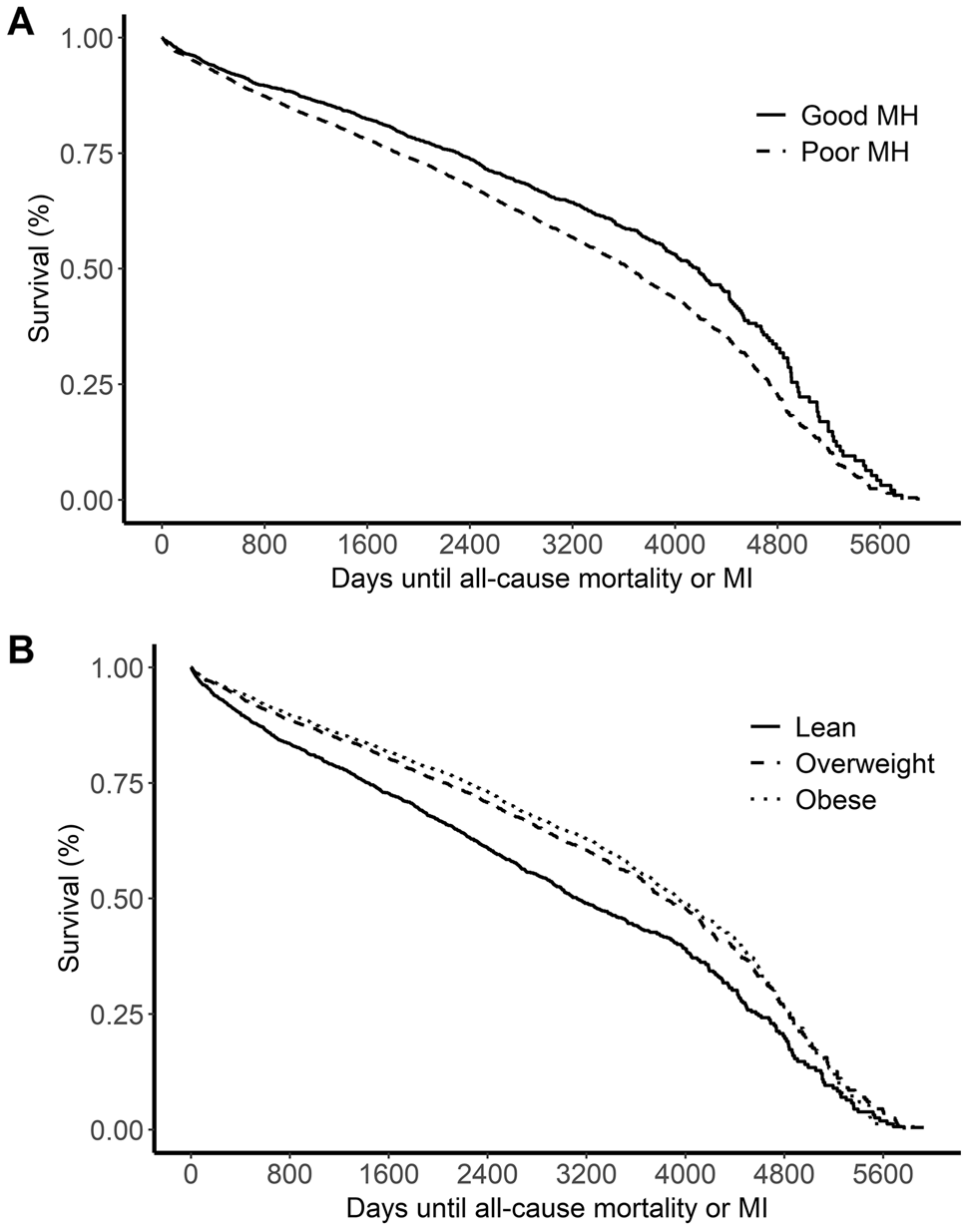
Unadjusted Kaplan–Meier plots for relationship between time-to-death or incident MI and metabolic health (**A**) and BMI category (**B**).

There were 3845 death or MI events (44.3% of individuals; 6.5% MI, 37.8% deaths) with a mean time to event of 2433 days (SD 1263 days). Traditionally defined poor MH was associated with time-to-death/MI in the overall cohort (HR 1.38, 95% CI 1.28–1.49, p = 5.0 × 10^−17^, Fig. 1A). In the same models stratified by BMI category, traditionally defined poor MH was associated with a stepwise increase in HR for increasing BMI category: lean: HR 1.23, 95% CI 1.08–1.39, *p* = 1.4 × 10^−3^; overweight: HR 1.45, 95% CI 1.28–1.64, *p* = 7.2 × 10^−9^; obese: HR 1.53, 95% CI 1.32–1.77, *p* = 2.0 × 10^−8^. 1028 events occurred in the lean population (54% of lean individuals), with 411 events occurring in lean individuals with traditionally defined good MH.

Interestingly, after accounting for smoking history, obesity was protective for events, with higher BMI categories associated with a lower risk of events (overweight: HR: 0.71, 95% CI 0.65–0.77; obese: HR 0.65, 95% CI 0.59–0.70, both compared to referent lean BMI category) (Fig. 1B).

### Novel plasma biomarkers predict incident events independent of traditionally defined poor MH and across all BMI categories

To understand the prognostic capability of novel plasma biomarkers independent of traditional metabolic health parameters, we analyzed Cox models for each plasma biomarker with time-to-death/MI, adjusting for traditionally defined poor MH (Table 2). In these analyses, each plasma biomarker was significantly associated with time-to-death/MI in the overall cohort; GlycA and SCDA were associated with decreased survival, while LDL-P, LP-IR, HMSP and BCAA were all associated with increased survival. In analyses stratified by BMI category, each individual plasma biomarker remained significantly predictive of death/MI and with similar effect sizes across each BMI category except BCAA, which was associated with time-to-event only in obese individuals. Models for plasma biomarkers LDL-P, LP-IR, GlycA, and HMSP included 8385 individuals, and those for BCAA and SCDA included 3591 individuals.

**Table 2:** Hazard ratios, confidence intervals, and *p* values for death or incident MI prediction for traditionally defined poor MH and novel plasma biomarkers in the overall cohort and stratified by BMI category.

Model*	Overall cohort		Lean		Overweight		Obese	
	HR (95% CI)	*p* value	HR (95% CI)	*p* value	HR (95% CI)	*p* value	HR (95% CI)	*p* value
**Unadjusted model**								
MH	1.38 (1.28–1.49)	4.5 × 10^−17^	1.23 (1.08–1.39)	1.4 × 10^−3^	1.45 (1.28–1.64)	7.2 × 10^−9^	1.53 (1.32–1.77)	2.0 × 10^−8^
MH + LDL-P	0.91 (0.88–0.94)	2.5 × 10^−8^	0.91 (0.85–0.97)	5.3 × 10^−3^	0.88 (0.83–0.94)	3.3 × 10^−5^	0.93 (0.88–0.98)	8.1 × 10^−3^
MH + LP-IR	0.78 (0.75–0.81)	3.1 × 10^−40^	0.75 (0.70–0.82)	3.3 × 10^−12^	0.79 (0.74–0.84)	2.5 × 10^−14^	0.79 (0.75–0.83)	1.3 × 10^−17^
MH + GlycA	1.27 (1.24–1.31)	2.9 × 10^−59^	1.28 (1.21–1.35)	6.2 × 10^−19^	1.32 (1.26–1.39)	2.8 × 10^−27^	1.23 (1.17–1.29)	2.4 × 10^−16^
MH + HMSP	0.66 (0.64–0.68)	3.6 × 10^−123^	0.67 (0.63–0.72)	9.6 × 10^−34^	0.63 (0.60–0.67)	1.4 × 10^−49^	0.67 (0.63–0.71)	1.1 × 10^−44^
MH + BCAA	0.91 (0.86–0.96)	3.3 × 10^−4^	0.94 (0.85–1.04)	0.23	0.93 (0.85–1.02)	0.13	0.87 (0.80–0.94)	6.3 × 10^−4^
MH + SCDAs	1.25 (1.20–1.30)	5.1 × 10^−29^	1.15 (1.06–1.24)	6.9 × 10^−4^	1.3 (1.23–1.38)	3.7 × 10^−19^	1.31 (1.22–1.41)	2.5 × 10^−13^
Fully adjusted model**								
MH + clinical covariates**	1.21 (1.11–1.31)	5.3 × 10^−6^	1.04 (0.9–1.19)	0.61	1.22 (1.07–1.40)	3.6 × 10^−3^	1.53 (1.3–1.81)	4.4 × 10^−7^
MH + LDL-P + clinical covariates**	0.8 (0.73–0.87)	7.2 × 10^−7^	0.72 (0.6–0.87)	4.6 × 10^−4^	0.71 (0.61–0.83)	1.5 × 10^−5^	0.97 (0.84–1.12)	0.66
MH + LP-IR + clinical covariates**	0.83 (0.80–0.87)	1.1 × 10^−18^	0.8 (0.73–0.87)	8.7 × 10^−7^	0.84 (0.78–0.90)	4.6 × 10^−7^	0.84 (0.78–0.89)	3.2 × 10^−8^
MH + GlycA + clinical covariates**	1.27 (1.23–1.31)	1.6 × 10^−49^	1.25 (1.18–1.33)	9.1 × 10^−14^	1.3 (1.24–1.38)	1.1 × 10^−21^	1.24 (1.18–1.31)	2.0 × 10^−16^
MH + HMSP + clinical covariates**	0.71 (0.68–0.74)	1.4 × 10^−70^	0.68 (0.63–0.73)	2.7 × 10^−24^	0.71 (0.66–0.76)	2.2 × 10^−25^	0.72 (0.68–0.77)	4.0 × 10^−25^
MH + BCAA + clinical covariates**	0.94 (0.89–1.00)	0.051	0.92 (0.81–1.03)	0.14	0.97 (0.88–1.08)	0.62	0.95 (0.87–1.04)	0.29
MH + SCDAs + clinical covariates**	1.24 (1.14–1.34)	9.2 × 10^−7^	1.23 (1.06–1.41)	4.7 × 10^−3^	1.36 (1.19–1.55)	6.2 × 10^−6^	1.05 (0.87–1.28)	0.61

*MH models included 8671 samples; LDL-P, LP-IR, GlycA, and HMSP models included 8385 samples; BCAA and SCDA models included 3591 samples.

**Clinical covariates include: age, sex, race, LVEF, CAD, family history of CAD, smoking, LDL-C, and creatinine.

Sensitivity analyses including individuals with medication data (N = 3,174 out of 8761 [36.2%]) were conducted including statin use as a binary covariable (Supplementary Table 1). Overall, the magnitude and significance of results remained the same in these analyses.

In a full Cox model inclusive of traditionally defined poor MH and all plasma biomarkers, each individual plasma biomarker remained independently significant with a similar magnitude of effect in the overall cohort (Supplementary Table 2). In similar full models stratified by BMI category, each plasma biomarker other than LP-IR and LDL-P also remained significantly predictive of events in one or more obesity categories, with GlycA, HMSP, and SCDA maintaining significance across all BMI categories (Supplementary Table 2).

The proportional hazards assumption was violated in our models for plasma biomarkers LP-IR, GlycA, and HMSP in the overall population and the BMI-stratified analyses. To examine the potential impact of non-proportional hazards related to these plasma biomarkers, we conducted sensitivity analyses allowing for a time-varying coefficient in the model. All plasma biomarkers were still significantly associated with events in all tested models (*p* < 10^−12^). Estimates of the HR at various timepoints are given in Supplementary Table 3; these demonstrate that the magnitude of the estimated HR for each plasma biomarker increases when the time-varying coefficient is included in the model, compared to the results in Table 2. Therefore, for simplicity in the remaining analyses and with the understanding that this may lead to an underestimation of the effect sizes for LP-IR, GlycA, and HMSP, we utilized a Cox model without time-varying coefficients.

### Novel plasma biomarkers independently predict events in full multivariable models additionally adjusted for clinical risk factors

While our primary goal was to understand novel plasma biomarkers as markers of metabolic health using event prediction as a benchmark for assessing importance, there are also other known clinical risk factors for incident CVD events that are not captured by models of traditionally defined poor MH; thus, we also created full multivariable models, including age, sex, race, LVEF, smoking, LDL-C, creatinine, and presence/absence of CAD. In these models, which also adjusted for traditionally defined poor MH, each individual novel plasma biomarker except BCAA remained predictive of events in the overall population (Table 2). In BMI category stratified analyses, LP-IR, GlycA, and HMSP plasma biomarkers were significantly and consistently associated with incident CVD events across all BMI categories with a similar magnitude of effect in each category, suggesting they are important biomarkers regardless of BMI. Traditionally defined poor MH remained predictive overall and in the overweight and obese categories, but was not significant in its prediction of events in the lean group (Table 2).

In full multivariable models inclusive of traditionally defined poor MH, CVD clinical risk factors, and all plasma biomarkers, four plasma biomarkers were significant in the overall cohort: GlycA (HR 1.23, 95% CI 1.17–1.29, *p* = 3.6 × 10^−18^); HMSP (HR 0.76, 95% CI 0.71–0.81, *p* = 2.0 × 10^−18^); BCAA (HR 0.93, 95% CI 0.88–0.99, *p* = 0.01); and SCDA (HR 1.17, 95% CI 1.08–1.28, *p* = 2.7 × 10^−4^). In analyses stratified by BMI category, only GlycA and HMSP remained significantly predictive of events across all BMI categories (Supplementary Table 4).

### Novel plasma biomarkers incrementally improve CVD event prediction

To further assess the incremental predictive capabilities of each novel plasma biomarker, we compared models using the AIC and AUC. These models demonstrated modest improvement of model fit with addition of any individual novel plasma biomarker to traditionally defined poor MH in the overall cohort: traditionally defined poor MH (AIC 20,240, AUC 0.58), LDL-P (AIC 20,222, AUC 0.59), LP-IR (AIC 20,179, AUC 0.61), GlycA (AIC 20,162, AUC 0.63), HMSP (AIC 20,051, AUC 0.64), BCAA (AIC 20,232, AUC 0.61), SCDA (AIC 20,155, AUC 0.62) (Table 3). BMI stratified categories showed similar improvement of model fit with addition of any individual novel plasma biomarker other than LDL-P (Table 3). When all plasma biomarkers are included, there is greater improvement in model fit than with any single variable alone in the overall cohort (AIC 19,919, AUC 0.69) and within each BMI category (Table 3).

**Table 3:** Comparison of model fit characteristics overall and stratified by BMI category.

Model^a^	Overall		Lean		Overweight		Obese	
	AIC*	AUC	AIC*	AUC	AIC*	AUC	AIC*	AUC
**Without clinical covariates**								
MH	20,240	0.58	4343	0.55	5846	0.56	7038	0.54
MH + LDL-P	20,222	0.59	4343	0.55	5837	0.57	7032	0.55
MH + LP-IR	20,179	0.61	4332	0.59	5831	0.60	7006	0.59
MH + GlycA	20,162	0.63	4315	0.62	5821	0.63	7016	0.60
MH + HMSP	20,051	0.64	4300	0.63	5782	0.65	6952	0.62
MH + BCAA	20,232	0.61	4344	0.57	5846	0.61	7032	0.59
MH + SCDA	20,155	0.62	4329	0.57	5807	0.62	7010	0.59
MH + all plasma biomarkers	19,919	0.69	4269	0.68	5745	0.69	6907	0.67
With clinical covariates*								
MH + clinical covariates	19,676	0.75	4241	0.71	5645	0.76	6780	0.75
MH + LDL-P + clinical covariates	19,675	0.75	4243	0.72	5643	0.76	6782	0.75
MH + LP-IR + clinical covariates	19,660	0.75	4239	0.72	5644	0.76	6771	0.75
MH + GlycA + clinical covariates	19,599	0.76	4210	0.74	5619	0.78	6761	0.76
MH + HMSP + clinical covariates	19,561	0.76	4207	0.73	5613	0.77	6728	0.76
MH + BCAA + clinical covariates	19,674	0.75	4241	0.72	5647	0.76	6781	0.75
MH + SCDA + clinical covariates	19,651	0.75	4236	0.72	5630	0.76	6781	0.75
MH + all plasma biomarkers + clinical covariates	19,482	0.77	4180	0.75	5588	0.78	6711	0.77

*AIC analyses were restricted to individuals who had data needed for all models of traditionally defined MH and for all biomarkers (n = 3222).

Clinical covariates include: age, sex, race, LVEF, CAD, family history of CAD, smoking, LDL-C, and creatinine.

In models inclusive of clinical covariates, we found modest improvement of model performance beyond that of traditionally defined poor MH, with further improvement in performance with addition of any individual plasma biomarker other than LDL-P in the overall cohort: traditionally defined poor MH (AIC 19,674, AUC 0.74), LDL-P (AIC 19,673, AUC 0.74), LP-IR (AIC 19,658, AUC 0.74), GlycA (AIC 19,598, AUC 0.75), HMSP (AIC 19,560, AUC 0.75), BCAA (AIC 19,672, AUC 0.75), SCDA (AIC 19,649, AUC 0.75) (Table 3). BMI stratified categories showed improvement of model fit with addition of GlycA, HMSP, BCAA, and SCDA. Similar to prior, the greatest improvement in model performance occurs in the model inclusive of traditionally defined poor MH, all plasma biomarkers, and clinical covariates (AIC 19,481, AUC 0.77) (Table 3).

We also explored the potential association between the plasma biomarkers and family history of CAD (Supplementary Table 5) and found significant correlations between LP-IR (β = 0.20, *p* = 1.18 × 10^−17^), LDLP (β = 0.07, *p* = 0.002) and HMSP (β = 0.12, *p* = 7.49 × 10^−7^) in the overall cohort. In the lean group, only LP-IR (β = 0.25, *p* = 1.82 × 10^−8^) and HMSP (β = 0.16, *p* = 0.002) were associated with family history of CAD.

## Discussion

In this large cardiovascular cohort study, we demonstrate that novel plasma biomarkers discovered through prior studies of CVD may serve as better markers of poor metabolic health as determined by their ability to predict risk of adverse cardiovascular events across all BMI categories, independent of, and incremental to, traditionally defined poor MH parameters. Our most striking results include: (1) traditionally defined poor MH does not predict CVD events in lean individuals in full multivariable models, but the majority of novel plasma biomarkers do; (2) GlycA and HMSP plasma biomarkers had strong predictive capabilities in the overall cohort and across all BMI categories even after adjusting for traditionally defined poor MH and other clinical risk factors, and with the greatest improvements in AUC on top of traditionally defined poor MH; (3) adding all novel plasma biomarkers to traditionally defined poor MH improved the AUC from 0.58 to 0.69 in the overall cohort, and with a similar magnitude of improvement in lean and overweight BMI categories where metabolic health is perhaps the most heterogeneous.

In our study, 55.3% of lean individuals met criteria for poor MH, while 18.5% of obese individuals met criteria for having good MH. Furthermore, of the 3845 total events, 24.3% occurred in individuals with good MH and 26.7% occurred in lean individuals. These highlight the disconnect between traditionally defined poor MH, weight, and risk of morbidity and mortality.

We used hard outcomes of death or incident MI to determine utility of traditional and novel risk factors. Overall, all of the novel plasma biomarkers assessed in this study other than BCAA were found to be predictive of incident CVD events, even after adjusting for traditionally defined poor MH and additional clinical risk factors. This predictive capability held true in analyses stratified by BMI category for plasma biomarkers LP-IR, GlycA, and HMSP. Furthermore, GlycA and HMSP were strongly predictive of events in the lean group, which is classically felt to be of lesser adverse cardiovascular event risk but may actually have a higher risk. Importantly, novel plasma biomarkers also improved model fit on top of traditionally defined poor MH and additional clinical risk factors, suggesting that they have incremental risk-predictive capabilities beyond what is easily assessed clinically.

Serum concentrations of GlycA, a novel inflammatory biomarker that is an aggregate measure of enzymatically glycosylated, acute-phase proteins, have been shown to independently predict incident CVD events in the MESA study (HR 1.33, 95% CI 1.24–1.42) and in the JUPITER trial (HR 1.33, 95% CI 1.21–1.45)^29,30^, and with presence and extent of CAD^17^. The biologic relevance of elevations in GlycA seen in these studies may be due to effects on glycosylation of HDL components resulting in impaired function^17^.

In full multivariate models, a factor composed of SCDA was robust for prediction of death or MI in the overall cohort as well as in lean and overweight individuals. These plasma biomarkers have been shown to be associated with increased risk of adverse CVD related events in the CATHGEN cohort^19,20^, and appear to be reporting on dysregulated endoplasmic reticulum stress^28,31^.

The role of HDL cholesterol in CVD is complex; studies have demonstrated an inverse relationship of HDL-C with CVD, but pharmacologic intervention to increase HDL-C have failed to show improved outcomes^32^. A growing number of studies, however, are showing that NMR-derived lipoprotein particle number more closely correlates with risk of adverse CVD outcomes than traditional LDL-C and HDL-C measures^18,33^. The cardioprotective effects of HMSP seen in the current study likely depend on specific HDL subparticle characteristics that are not well accounted for by traditional HDL-C, which is preferentially affected by larger, cholesterol rich particles^32^; similar to that seen in a recent study showing that the sum of medium- and small-size HDL particles was the strongest predictor of mortality^18^.

In the current study, higher LDL-P levels were protective against death or MI. From prior work with the CATHGEN population, we have seen evidence of a similar lipid paradox^13–15,20^. The etiology of this phenomenon remains incompletely elucidated and may be related to medication use (e.g. patients with higher LDL levels are treated earlier and for longer with statins) but new evidence might suggest that nutritional status plays a key role^34^.

Unexpectedly, while HOMA-IR was higher with increasing BMI, higher LP-IR levels were protective of death or MI. This might be related to the observed obesity paradox in our secondary prevention dataset, i.e. because obesity is protective of adverse outcomes and obese individuals have higher HOMA-IR.

In addition, individuals with poor metabolic health had a higher prevalence of a positive family history of CAD as compared with individuals with good metabolic health which was also true in the overweight category. However, there were no significant differences in the lean and obese group, suggesting that this more simple screening tool would not be sufficient for defining CVD risk. Moreover, we found correlations between LP-IR, LDLP and HMSP and family history of CAD in the overall cohort. In the lean group, only LP-IR and HMSP were associated with family history of CAD. This is as predicted given the known heritability of these markers.

Regarding the clinical utility of our findings and testing for these plasma biomarkers, it is relatively quick, reproducible, and of course, by their nature, non-invasive^35^. Also, mass spectrometry is currently already in use for new-born screening and more severe metabolic phenotypes (e.g. phenylketonuria, maple-syrup urine disease, etc.). Although the use of some of these plasma biomarkers is still in its infancy, there is a growing body of evidence that supports their clinical utility in cardiovascular disease^35^. Of note, the NMR-derived lipoproteins are commonly used in clinical practice already.

Several strengths of our study deserve mention including the relatively large sample size, availability and length of follow-up data for hard CVD outcomes, fasting samples collected under a rigorous and similar protocol, and the breadth of clinical variables available. Importantly, many prior studies of metabolic health measures do not have a “gold standard” to tag improvement of models to; the use of a clinically relevant outcome of death or MI is an important metric that we were able to use to compare models. However, since this is a cross-sectional, observational study, we were unable to account for chronic dietary and environmental variations in plasma biomarker levels, nor did we have sufficient medication data to enable adjustment for medication use (although previous work showed such adjustments had minimal influence on plasma biomarker levels)^27^. Finally, although the model of metabolic health improved with the addition of clinical covariates to the plasma biomarkers, the increase was modest, which suggests that clinical covariates contribute more to the models rather than the biomarkers. However, we note that the greatest improvement was seen in the lean group, a group that is often dismissed clinically as having low CVD risk, and as such, biomarkers may have the greatest clinical utility for identifying higher risk individuals in this group.

In conclusion, with growing evidence of the discordance between metabolic health and BMI, it is crucial to improve clinical models for early recognition and characterization of individuals at high risk for CVD events, and thus at greatest need for early intensive therapeutic intervention. We found plasma biomarkers had the potential to improve clinical models, to better classify overall metabolic health, and to independently predict events, irrespective of obesity stratum. This was especially striking given that traditionally defined MH models were unable to predict events in the lean group, while novel plasma biomarkers were successful in prediction of events; this suggests that novel plasma biomarkers may be better markers of metabolic health in this patient population than current standard clinical models.

## Supplementary information

Supplementary information.
