# Supplementary material for: Novel plasma biomarkers improve discrimination of metabolic health independent of weight
Source: Sci Rep. 2020 Dec 7;10:21365. doi: 10.1038/s41598-020-78478-w (PMC7721699; doi:10.1038/s41598-020-78478-w)
Supplement: Supplementary file 1 — Supplementary information. [file 41598_2020_78478_MOESM1_ESM.docx]

**Supplementary Tables**

**Novel Plasma Biomarkers Improve Discrimination of Metabolic Health Independent of Weight**

Stephen Ellison^1*^, Jawan W. Abdulrahim^2*^, Lydia Coulter Kwee^2^, Nathan A. Bihlmeyer^2^, Neha Pagidipati^3^, Robert McGarrah^2,3^, James R. Bain^2^, William E. Kraus^2,3^, Svati H. Shah^2,3^*

^1^Department of Anesthesiology, Duke University Medical Center, Durham, NC;

^2^Duke Molecular Physiology Institute, Duke University School of Medicine, Durham, NC;

^3^Division of Cardiology, Department of Medicine, Duke University School of Medicine, Durham, NC

*Contributed equally

*Corresponding author:

Svati H. Shah, MD, MS, MHS

300 North Duke St.

Durham, NC, 27701

Phone: 919-684-1808

Fax: 919-684-8907

[svati.shah@duke.edu](mailto:svati.shah@dm.duke.edu)

**Supplemental Table S1.** Sensitivity analysis on individuals with statin data (N=3,174) available with Hazard ratios, confidence intervals, and p-values for death or incident MI prediction for traditionally defined poor MH and novel plasma biomarkers in full multivariable models (inclusive of clinical covariates and statin use as a binary covariate) in the overall cohort and stratified by BMI category.

|  | **Overall cohort** | | | **Lean** | | | **Overweight** | | | **Obese** | | |
| --- | --- | --- | --- | --- | --- | --- | --- | --- | --- | --- | --- | --- |
| **Model** | **HR** | **95% CI** | **p value** | **HR** | **95% CI** | **p value** | **HR** | **95% CI** | **p value** | **HR** | **95% CI** | **p value** |
| **MH** | 1.11 | 0.98-1.27 | 0.11 | 1 | 0.81-1.25 | 0.97 | 1.09 | 0.87-1.36 | 0.45 | 1.46 | 1.11-1.94 | 7.6x10^-3^ |
| **MH + LDL-P** | 0.75 | 0.65-0.88 | 2.8x10^-4^ | 0.74 | 0.55-1.01 | 0.054 | 0.68 | 0.52-0.89 | 5.2x10^-3^ | 0.81 | 0.64-1.03 | 0.092 |
| **MH + LP-IR** | 0.78 | 0.73-0.84 | 3.5x10^-12^ | 0.78 | 0.68-0.9 | 8.7x10^-4^ | 0.79 | 0.7-0.89 | 1.4x10^-4^ | 0.74 | 0.66-0.82 | 8.0x10^-8^ |
| **MH + GlycA** | 1.28 | 1.21-1.35 | 2.1x10^-17^ | 1.25 | 1.14-1.37 | 2.3x10^-6^ | 1.35 | 1.22-1.49 | 8.9x10^-9^ | 1.23 | 1.11-1.36 | 7.3x10^-5^ |
| **MH + HMSP** | 0.67 | 0.63-0.71 | 3.4x10^-36^ | 0.68 | 0.6-0.77 | 2.7x10^-10^ | 0.62 | 0.56-0.7 | 3.5x10^-16^ | 0.69 | 0.63-0.76 | 4.1x10^-13^ |
| **MH + BCAA** | 0.94 | 0.85-1.05 | 0.28 | 0.91 | 0.74-1.12 | 0.36 | 1.06 | 0.88-1.27 | 0.56 | 0.83 | 0.69-1.01 | 0.058 |
| **MH + SCDAs** | 1.45 | 1.23-1.71 | 1.2x10^-5^ | 1.26 | 0.98-1.63 | 0.07 | 1.78 | 1.37-2.32 | 1.8x10^-5^ | 1.22 | 0.88-1.68 | 0.22 |

**Supplemental Table S2.** Presented are hazard ratios, confidence intervals, and p-values for death or incident MI prediction in a model including all plasma biomarkers and adjusting for traditionally defined poor MH in the overall cohort and stratified by BMI category.

|  | **Overall Cohort** | | | **Lean** | | | **Overweight** | | | **Obese** | | |
| --- | --- | --- | --- | --- | --- | --- | --- | --- | --- | --- | --- | --- |
| **Metabolic**  **Biomarker*** | **HR** | **95% CI** | **p value** | **HR** | **95% CI** | **p value** | **HR** | **95% CI** | **p value** | **HR** | **95% CI** | **p value** |
| **LDL-P** | 0.93 | 0.88-0.98 | 0.01 | 0.94 | 0.85-1.04 | 0.22 | 0.91 | 0.82-1.00 | 0.05 | 0.93 | 0.86-1.01 | 0.10 |
|  |  |  |  |  |  |  |  |  |  |  |  |  |
| **LP-IR** | 0.92 | 0.87-0.99 | 0.02 | 0.91 | 0.78-1.05 | 0.20 | 0.93 | 0.83-1.04 | 0.19 | 0.93 | 0.84-1.02 | 0.12 |
|  |  |  |  |  |  |  |  |  |  |  |  |  |
| **GlycA** | 1.24 | 1.19-1.29 | 1.9x10^-21^ | 1.28 | 1.17-1.39 | 1.7x10^-8^ | 1.23 | 1.14-1.33 | 1.5x10^-7^ | 1.22 | 1.14-1.31 | 3.0x10^-8^ |
|  |  |  |  |  |  |  |  |  |  |  |  |  |
| **HMSP** | 0.75 | 0.71-0.79 | 8.6x10^-23^ | 0.79 | 0.71-0.88 | 2.0x10^-5^ | 0.75 | 0.67-0.83 | 6.7x10^-8^ | 0.69 | 0.63-0.76 | 1.3x10^-13^ |
|  |  |  |  |  |  |  |  |  |  |  |  |  |
| **BCAA** | 0.94 | 0.89-0.99 | 0.02 | 0.93 | 0.84-1.04 | 0.20 | 1.00 | 0.91-1.1 | 0.97 | 0.88 | 0.81-0.96 | 4.6x10^-3^ |
|  |  |  |  |  |  |  |  |  |  |  |  |  |
| **SCDA** | 1.19 | 1.13-1.24 | 3.2x10^-12^ | 1.22 | 1.09-1.37 | 5.7x10^-4^ | 1.19 | 1.11-1.27 | 2.1x10^-6^ | 1.15 | 1.06-1.25 | 7.4x10^-4^ |
|  |  |  |  |  |  |  |  |  |  |  |  |  |
| **All metabolic  biomarkers** | 1.41 | 1.23-1.61 | 6.7x10^-7^ | 1.2 | 0.95-1.52 | 0.12 | 1.5 | 1.19-1.89 | 5.4x10^-4^ | 1.65 | 1.27-2.14 | 1.8x10-4 |

*MH models included 8671 samples; LDL-P, LP-IR, GlycA, and HMSP models included 8385 samples; BCAA and SCDA models included 3591 samples.

**Supplemental Table S3.** Presented are estimates of the hazard ratios and p-value at various timepoints for death or incident MI prediction for those plasma biomarkers with a deviation of the assumptions of proportional hazards in the overall cohort and stratified by BMI category.

|  |  | **Estimated Hazard Ratios** | | | | |  |
| --- | --- | --- | --- | --- | --- | --- | --- |
|  | **Metabolic**  **Biomarker*** | **6 months** | **1 year** | **3 years** | **5 years** | **10 years** | **p-value** |
| **Overall Cohort** | **LP-IR** | 0.43 | 0.45 | 0.49 | 0.51 | 0.54 | <10^-16^ |
|  | **GlycA** | 2.22 | 2.11 | 1.95 | 1.88 | 1.79 | <10^-16^ |
|  | **HMSP** | 0.28 | 0.30 | 0.34 | 0.36 | 0.39 | <10^-16^ |
| **Lean** | **LP-IR** | 0.47 | 0.49 | 0.53 | 0.54 | 0.57 | 1.3x10^-12^ |
|  | **GlycA** | 1.85 | 1.79 | 1.69 | 1.65 | 1.60 | <10^-16^ |
|  | **HMSP** | 0.23 | 0.25 | 0.29 | 0.32 | 0.35 | <10^-16^ |
| **Overweight** | **LP-IR** | 0.40 | 0.43 | 0.47 | 0.49 | 0.52 | 4.4x10^-16^ |
|  | **GlycA** | 2.87 | 2.68 | 2.40 | 2.28 | 2.12 | <10^-16^ |
|  | **HMSP** | 0.28 | 0.30 | 0.34 | 0.36 | 0.39 | <10^-16^ |
| **Obese** | **LP-IR** | 0.50 | 0.52 | 0.56 | 0.57 | 0.60 | <10^-16^ |
|  | **GlycA** | 2.13 | 2.03 | 1.87 | 1.81 | 1.72 | <10^-16^ |
|  | **HMSP** | 0.34 | 0.36 | 0.40 | 0.42 | 0.44 | <10^-16^ |

*LP-IR, GlycA, and HMSP models included 8385 samples

**Supplemental Table S4.** Presented are hazard ratios, confidence intervals, and p-values for death or incident MI prediction in a full multivariable model including all plasma biomarkers and adjusting for traditionally defined poor MH and CVD clinical risk factors in the overall cohort and stratified by BMI category.

|  | **Overall Cohort** | | | **Lean** | | | **Overweight** | | | **Obese** | | |
| --- | --- | --- | --- | --- | --- | --- | --- | --- | --- | --- | --- | --- |
| **Metabolic**  **Biomarker*** | **HR** | **95% CI** | **p value** | **HR** | **95% CI** | **p value** | **HR** | **95% CI** | **p value** | **HR** | **95% CI** | **p value** |
| **LDL-P^a^** | 1.00 | 0.85-1.17 | 0.98 | 1.00 | 0.71-1.4 | 0.98 | 0.87 | 0.66-1.15 | 0.33 | 1.25 | 0.96-1.61 | 0.09 |
|  |  |  |  |  |  |  |  |  |  |  |  |  |
| **LP-IR** | 0.99 | 0.91-1.07 | 0.79 | 0.98 | 0.82-1.18 | 0.86 | 1.06 | 0.93-1.21 | 0.41 | 0.90 | 0.80-1.02 | 0.11 |
|  |  |  |  |  |  |  |  |  |  |  |  |  |
| **GlycA** | 1.23 | 1.17-1.29 | 3.6x10^-18^ | 1.29 | 1.18-1.41 | 1.9x10^-8^ | 1.22 | 1.13-1.33 | 1.3x10^-6^ | 1.2 | 1.11-1.30 | 2.3x10^-6^ |
|  |  |  |  |  |  |  |  |  |  |  |  |  |
| **HMSP** | 0.76 | 0.71-0.81 | 2.0x10^-18^ | 0.75 | 0.66-0.85 | 3.2x10^-6^ | 0.78 | 0.69-0.87 | 6.2x10^-6^ | 0.72 | 0.65-0.80 | 3.0x10^-10^ |
|  |  |  |  |  |  |  |  |  |  |  |  |  |
| **BCAA** | 0.93 | 0.88-0.99 | 0.01 | 0.89 | 0.79-1.00 | 0.06 | 0.97 | 0.88-1.07 | 0.52 | 0.92 | 0.84-1.01 | 0.08 |
|  |  |  |  |  |  |  |  |  |  |  |  |  |
| **SCDA** | 1.17 | 1.08-1.28 | 2.7x10^-4^ | 1.18 | 0.99-1.42 | 0.07 | 1.23 | 1.09-1.40 | 9.7x10^-4^ | 1.04 | 0.85-1.26 | 0.72 |
|  |  |  |  |  |  |  |  |  |  |  |  |  |
| **All metabolic  biomarkers** | 1.10 | 0.96-1.27 | 0.18 | 0.93 | 0.72-1.19 | 0.56 | 1.15 | 0.90-1.46 | 0.27 | 1.39 | 1.05-1.82 | 0.02 |

*MH models included 8671 samples; LDL-P, LP-IR, GlycA, and HMSP models included 8385 samples; BCAA and SCDA models included 3591 samples.

Clinical covariates include: age, sex, race, LVEF, CAD, smoking, LDL-C, and creatinine.

**Supplemental Table S5.** Presented are correlation coefficients and p values for the correlations between family history of coronary artery disease and the plasma biomarkers.

|  | **All** | | **Lean** | | **Overweight** | | **Obese** | |
| --- | --- | --- | --- | --- | --- | --- | --- | --- |
| **Biomarker** | **β** | **p** | **β** | **p** | **β** | **p** | **β** | **p** |
| **LP-IR** | 0.20 | 1.18x10^-17^ | 0.25 | 1.82x10^-8^ | 0.15 | 5.87x10^-5^ | 0.18 | 5.27x10^-7^ |
| **GlycA** | -0.04 | 0.09 | -0.09 | 0.11 | -0.09 | 0.02 | 0.01 | 0.69 |
| **LDLP** | 0.07 | 2.4x10^-3^ | 0.08 | 0.10 | 0.00 | 0.94 | 0.11 | 3.1x10^-3^ |
| **HMSP** | 0.12 | 7.49x10^-7^ | 0.16 | 2.3x10^-3^ | 0.09 | 0.01 | 0.09 | 8.1x10^-3^ |
| **Factor 7** | 0.01 | 0.77 | -0.05 | 0.52 | 0.02 | 0.77 | 0.01 | 0.79 |
| **Factor 3** | -0.04 | 0.14 | 0.04 | 0.54 | -0.02 | 0.67 | -0.09 | 0.01 |
